# Validation of the Multiple Myeloma Symptom and Impact Questionnaire (MySIm-Q) in patients with multiple myeloma who were enrolled in the CARTITUDE-4 trial

**DOI:** 10.1186/s41687-026-01034-z

**Published:** 2026-03-13

**Authors:** Roberto Mina, Anne K. Mylin, Hisayuki Yokoyama, Hila Magen, Winfried Alsdorf, Leyla Shune, Iris Isufi, Simon J. Harrison, Urvi A. Shah, André De Champlain, Eva G. Katz, Katharine S. Gries, Jordan M. Schecter, Nikoletta Lendvai, Ana Slaughter, Carolina Lonardi, William Deraedt, Octavio Costa Filho, Nitin Patel, Erika Florendo, Kai Fai Ho, Lionel Karlin, Katja Weisel

**Affiliations:** 1 Department of Hematology and Medical Oncology, Winship Cancer Institute, Emory University, 1365 Clifton Rd N E, Atlanta, GA 30322 USA; 2 Department of Hematology, Rigshospitalet, Copenhagen, Denmark; 3 Tohoku University Graduate School of Medicine, Sendai, Japan; 4 Ramat-Gan, Faculty of Medicine, Tel Aviv University, Tel-Aviv, Israel; 5 University Medical Center Hamburg-Eppendorf, Hamburg, Germany; 6 The University of Kansas Medical Center, Kansas City, KS USA; 7 Department of Internal Medicine, Yale School of Medicine, Yale University, New Haven, CT USA; 8 Clinical Haematology, Peter MacCallum Cancer Centre and Royal Melbourne Hospital, Melbourne, Australia; 9 Sir Peter MacCallum Dep of Oncology, University of Melbourne, Parkville, Australia; 10 Memorial Sloan Kettering Cancer Center and Weill Cornell Medical College, New York, NY USA; 11 Johnson & Johnson, Horsham, PA USA; 12 Johnson & Johnson, Raritan, NJ USA; 13 Cilag GmbH International, Zug, Switzerland; 14 Johnson & Johnson, Buenos Aires, Argentina; 15 Johnson & Johnson, Beerse, Belgium; 16 Legend Biotech USA Inc., Somerset, NJ USA; 17 STAT-TU Inc, Elora, ON Canada; 18 Centre Hospitalier Lyon Sud, Pierre- Bénite, France; 19 The Myeloma Unit, Division of Hematology, University of Turin and Azienda Ospedaliero-Universitaria (AOU) Città della Salute e della Scienza di Torino, Turin, Italy

**Keywords:** Ciltacabtagene autoleucel, Multiple myeloma, Psychometric measurement properties, Patient-reported outcome instrument, MySIm-Q

## Abstract

**Background:**

The Multiple Myeloma Symptom and Impact Questionnaire (MySIm-Q) is a validated, disease-specific patient-reported outcome (PRO) instrument that measures symptoms and impacts experienced by patients with multiple myeloma (MM). We describe key measurement properties of the MySIm-Q instrument using CARTITUDE-4 data.

**Methodology:**

The phase 3 CARTITUDE-4 (NCT04181827) trial compares ciltacabtagene autoleucel with standard-of-care regimens in patients with lenalidomide-refractory MM after 1–3 lines of therapy. Following US Food and Drug Administration guidance on the use of PRO measures in clinical trials, the reliability, as well as aspects of construct validity, convergent and discriminant validity of the MySIm-Q symptom and impact scores (2 separate concepts) were assessed.

**Results:**

In total, 361 patients completed MySIm-Q assessments. Internal consistency results met the predefined threshold (McDonald’s ꞷ coefficient > 0.7; ꞷ=0.87 for total symptom scores, ꞷ=0.79 for total impact scores), while test-retest reliability was slightly below this threshold (intraclass correlation coefficient [ICC](2,1) > 0.70; ICC = 0.67 and 0.65, respectively). Known-groups validity of total symptom and total impact scores was established through multiple hypotheses. Factor scores estimated from the confirmatory factor analysis model were highly correlated with simple observed scores calculated in symptom and impact scores. Item-level convergent and discriminant validity was supported for all and nearly all items, respectively. Domain convergent and discriminant validity for total scores were largely met.

**Conclusions:**

These results demonstrate that the MySIm-Q yields reliable and valid scores based on a number of evidentiary sources, supporting its use as a fit-for-purpose PRO instrument in clinical outcome assessments for patients with MM.

**Trial registration:**

CARTITUDE-4: ClinicalTrials.gov ID: NCT04181827. Date of registration: 27 November 2019. URL: https://www.clinicaltrials.gov/study/NCT04181827.

**Supplementary Information:**

The online version contains supplementary material available at 10.1186/s41687-026-01034-z.

## Introduction

Multiple myeloma (MM) is a hematologic malignancy characterized by symptoms of bone pain, fatigue, and reduced physical and cognitive functioning [1–3]. The treatment of MM comprises multidrug regimens and is generally administered continuously until disease progression or intolerance due to adverse events. Disease progression and adverse events often result in poor health-related quality of life (HRQoL) in patients with MM [1–3]. Additionally, the degree of MM symptom severity is associated with HRQoL, with severely symptomatic patients reporting the greatest decline in global health status/quality of life (GHS/QoL), physical functioning, social functioning, and future perspectives regarding their health [1]. Long MM disease duration is also associated with large reductions in GHS/QoL [1]. Furthermore, in a European multicenter cohort study, patients with treatment-related symptoms and longer treatment durations had lower GHS/QoL and decreased physical and social functioning [1].

HRQoL in patients with MM can be assessed by using several widely available patient-reported outcome (PRO) instruments, including the European Organisation for Research and Treatment of Cancer quality of life questionnaire multiple myeloma module 20 (EORTC QLQ-MY20), the Functional Assessment of cancer therapy MM module, and the MD Anderson Symptom Inventory MM module [4–6]. These were developed as fit-for-purpose PRO tools or instruments that are sufficiently validated to support their context of use. However, development and validation of these instruments occurred prior to recent therapeutic advances in MM, such as monoclonal antibodies, chimeric antigen receptor T-cell therapies, and bispecific antibodies [7]. As such, current PRO instruments may not accurately reflect the disease experience of patients with MM who are undergoing treatment with recently approved therapies [8–10].

The Multiple Myeloma Symptom and Impact Questionnaire (MySIm-Q) is a newly developed MM-specific PRO instrument with a total symptom score and total impact score, designed to account for the changing treatment landscape in MM [11]. It addresses the limitations of legacy PRO instruments by measuring disease-related symptoms and impacts in patients receiving various MM therapies with differing mechanisms of action, and it can be used alongside other PRO instruments to measure and complement core PRO, efficacy, and tolerability endpoints in oncology clinical trials [11]. The MySIm-Q was developed by following best practice standards for patient-focused outcome measurements defined by the US Food and Drug Administration [12] and methods for establishing content validity recommended by the International Society for Pharmacoeconomics and Outcomes Research [11, 13, 14].

Evidence to support content validity [11] and preliminary assessment of MySIm-Q measurement properties using data from the phase 2, open-label, multicohort CARTITUDE-2 study of ciltacabtagene autoleucel (cilta-cel; NCT4133636) [15] and treatments reflective of the current treatment paradigm showed reliable and valid scores, for their intended use [16]. In these analyses, the MySIm-Q symptom score internal consistency was acceptable (Cronbach’s α-coefficient, 0.89) and test-retest reliability was supported (intraclass correlation coefficient [ICC(2,1)]), 0.81). MySIm-Q also demonstrated acceptable concurrent validity with existing European Organisation for Research and Treatment of Cancer quality of life questionnaire core 30 (EORTC QLQ-C30) symptom and impact measures, and known group validity showed that the MySIm-Q symptom score differentiated between disease severity groups [16]. These findings support the use of the MySIm-Q as a fit-for-purpose PRO instrument in MM clinical trials. The objective of this study was to assess measurement properties of the MySIm-Q using data from the larger phase 3, randomized, controlled CARTITUDE-4 study (NCT04181827) [17].

## Methods

### Study population in CARTITUDE-4

CARTITUDE-4 is a phase 3, randomized, open-label study. Detailed information on the study design and eligibility criteria have been previously published [17]. Briefly, patients with lenalidomide-refractory MM who received 1 to 3 prior lines of therapy (LOT), including a proteasome inhibitor (PI) and an immunomodulatory drug (IMiD), were assigned to receive either physician’s choice of standard care (SOC; pomalidomide, bortezomib, and dexamethasone [PVd], or daratumumab, pomalidomide, and dexamethasone [DPd]) or a single cilta-cel infusion. In the cilta-cel arm, patients underwent apheresis, followed by ≥ 1 cycle of bridging therapy (physician’s choice of PVd or DPd), lymphodepletion (fludarabine and cyclophosphamide), and a single cilta-cel infusion (any portion of the preceding sequence composed the study treatment) [17].

The trial was conducted in accordance with the principles of the Declaration of Helsinki and the International Conference on Harmonisation guidelines for Good Clinical Practice. An independent ethics committee or institutional review board at each site approved the protocol before study-related procedures commenced. All patients provided written informed consent before enrollment into the study.

### PRO instruments and assessment schedule

In addition to MySIm-Q, PRO instruments in CARTITUDE-4 included EORTC QLQ-C30 and patient global impression of MM symptom severity (PGIS). Details on PRO instruments, collection, and questionnaire assessment are provided in the Supplementary Information.

The MySIm-Q assessed patients’ experiences with MM and consisted of 17 items that were organized into eight domains comprising five symptom (pain, neuropathy, fatigue, digestive, and cognitive) and three impact (activity limitations, social functioning, and emotional impact) domains [11]. In total, 10 scores were derived (five scores for symptom domains, three scores for impact domains, and two scores for total symptom and impact scores). Total symptom score and a total impact score were calculated separately, with no single, total score. Scores for symptom and impact domains were computed by taking the average of ≥ 1 single items. Total scores were the average of symptom domain or impact domain scores. As weighting by item would give more weight to domains that have more items, the total scores were evenly weighted by domain rather than by item. The recall period was the past 7 days. Response options were scored on a five-point verbal rating scale, depending on severity (no [0], a little [1], moderate [2], quite a bit [3], severe/very much [4]) or frequency (never [0], rarely [1], some [2], most [3], always [4]) assessment, with higher scores representing greater symptom and impact burden.

PRO measurement collection during the first approximately 12 weeks of the study varied by study treatment based on study administration schedule differences. All questionnaires were administered using an electronic site-based tablet before clinical tests, procedures, or consultations to avoid influencing patients’ perception of their health state, and they were administered until disease progression.

### Statistical analysis

The intent-to-treat (ITT) population of CARTITUDE-4, defined as all patients who were randomly assigned to treatment, were included. Both treatment arms were pooled for all analyses except for the test-retest reliability analysis, which included patients from the SOC arm only.

Descriptive summary statistics for the MySIm-Q were reported as mean and standard deviations (SDs) and the frequency of response options for each item and scale score. Data were reported at time point 1 prior to treatment. Inter-item and domain correlations were estimated at time point 1 using Pearson, Spearman, and polychoric (inter-item redundancy only) correlation coefficients. Domain score skewness and kurtosis values (presence of asymmetry or outliers in item-level data) at time point 1 were examined for floor (i.e. high proportion of responses were at the lowest possible level) or ceiling (i.e. high proportion of responses were at the highest possible level) effects.

#### Reliability

Test-retest reliability (coefficient of stability) was assessed in clinically stable patients in the SOC arm using a two-way random variant intraclass correlation coefficient (ICC [2, 1]) per Shrout and Fleiss and convention [18]. ICC(2,1) values ≥ 0.70 were considered evidence of acceptable test-retest reliability. A retest interval of 3 to 4 weeks was used for the analysis. Sensitivity analyses were conducted in patients whose responses to the PGIS at cycle 1, day 1 and cycle 2, day 1 were identical. Internal consistency reliability was assessed using McDonald’s omega (ω) coefficient, with a value of > 0.70 supporting acceptable internal consistency reliability [19]. Error variances of items in single-item domains were fixed at 0.

#### Construct validity

A known-groups analysis of MySIm-Q scores by PGIS score using data at time point 1 was carried out to evaluate whether domain scores varied between groups of patients with known differences. The scores from patients in the following categories were compared: none/mild (reference), moderate, and severe/very severe. Categories were collapsed because of sample size. *P*-values were determined from two-sample *t*-tests comparing the means of each level of factor to the reference level of the factor.

A confirmatory factor analysis was conducted using data from time point 1 as a means of evaluating appropriateness of the prespecified total symptom and impact domain scores. Three models were analyzed. Model 1 was a unidimensional, first-order model with a single factor postulated for the symptom or impact domains model. Model 2 was a second-order model that was run separately for symptom and impact domains. The second-order factor was either symptoms or impacts, which loaded on five first-order symptom or three impact first-order factors. Model 3 was identical to Model 2 but had an additional constraint imposed, namely that factor loadings on symptoms or impacts were fixed as equal (tau-equivalent model).

#### Validity

Evidence of item convergent validity for the MySIm-Q was considered supportive if the correlation between each item and its adjusted hypothesized domain in the MySIm-Q questionnaire was > 0.40 [20]. Purified correlation coefficient values were estimated by removing an item from its domain and recalculating the value to control for spuriousness. Evidence of item discriminant validity for the MySIm-Q was supported if the correlation between each item and its hypothesized domain was higher than the correlation of that item with each of the other domains [20]. MySIm-Q symptom and impact domain convergent and discriminant validity evidence was collected by examining the relationship between scores on the EORTC QLC-C30 instrument that measured related/similar constructs or different constructs, respectively.

All analyses were performed using SAS version 9.4 or higher and confirmatory factor analysis measurements models were analyzed in SAS version 9.4 using PROC CALIS.

## Results

### Population

In CARTITUDE-4, a total of 419 patients were randomized to cilta-cel (*n* = 208) or SOC (*n* = 211 [PVd, *n* = 28; DPd, *n* = 183]), which composed the ITT population [17]. At the November 1, 2022, data cut-off date, median follow-up was 15.9 months. Baseline demographic and disease characteristics, efficacy, and safety data have been previously published [17]. MySIm-Q measurement properties were assessed in the 361 patients in the ITT population who had completed ≥ 1 assessment (58 patients did not complete ≥ 1 assessment).

### Descriptive analysis

Mean MySIm-Q item scores on a scale of 0 to 4 at time point 1 showed low symptom and impact burden, with scores ranging from 0.7 for poor appetite (SD 1.01; item 10) to 1.7 each for low energy (SD 1.05; item 6), tire easily (SD 1.04; item 7), and worry (SD 1.07; item 17). There was no strong evidence to suggest skewed or kurtotic item-level data (i.e. the presence of asymmetry or outliers in item-level data).

**Fig. 1:**
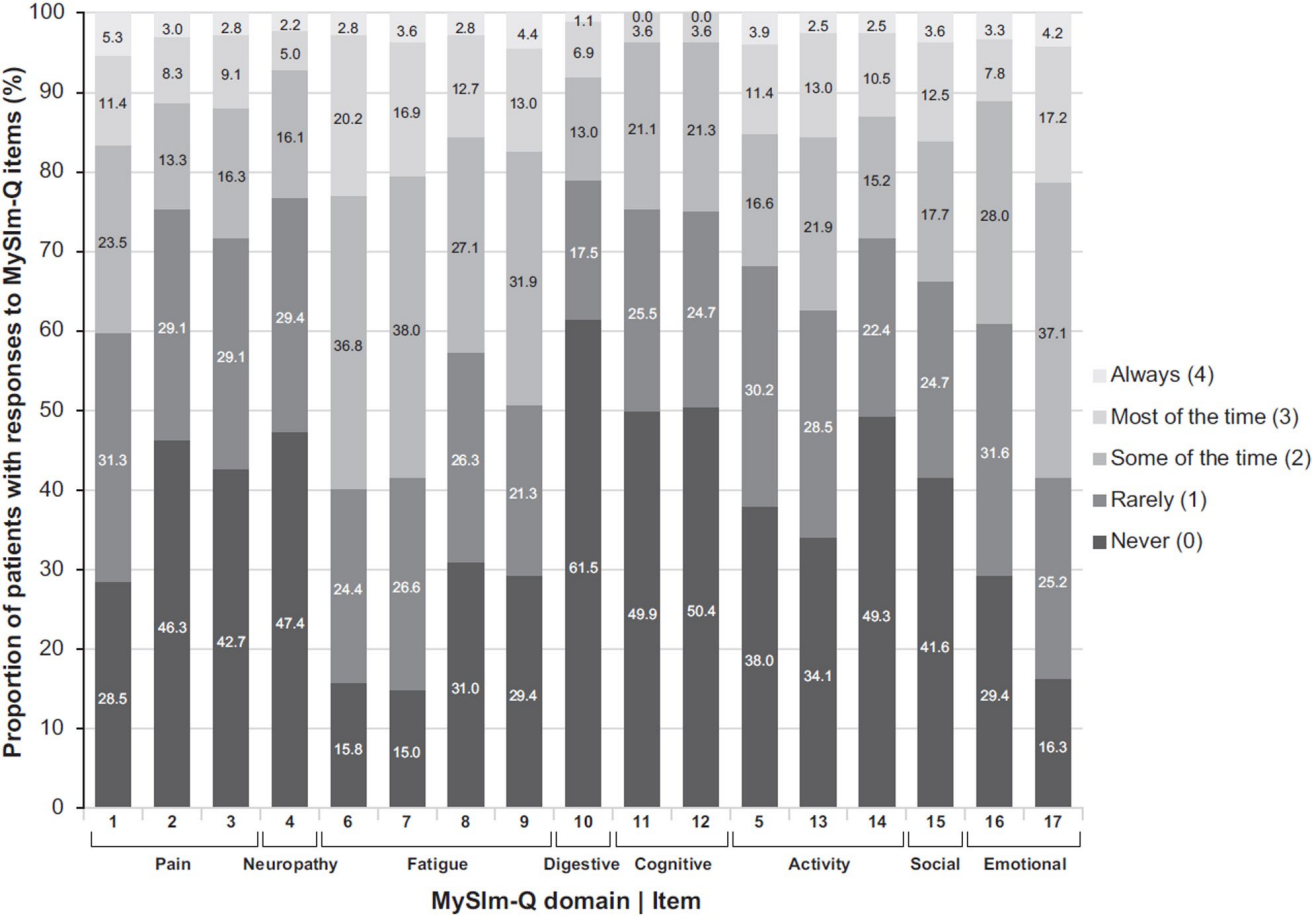
Descriptive summary of responses to MySIm-Q items/domains at time point 1. Items were scored from 0 to 4, with high scores representing greatest symptom and impact burden. MySIm-Q indicates Multiple Myeloma Symptom and Impact Questionnaire

The proportion of patients with responses to MySIm-Q items was > 0 for all but two response options at time point 1 (Fig. 1). No patient responded “always” to item 11 (difficulty with your memory) or item 12 (difficulty concentrating on things). These response options were selected by ≥ 1 patient at other time points in the clinical trial, so endorsement of every response option to each MySIm-Q item was established. High inter-item correlations at time point 1 were observed for having low energy (item 6) and tire easily (item 7), with a Pearson correlation of 0.79, Spearman correlation of 0.79, and a polychoric correlation of 0.85. Thus, there generally appeared to be little or no redundancy between items in the MySIm-Q.

Similar to mean MySIm-Q item scores, mean domain, total symptom, and total impact scores showed low symptom and impact burden; mean scores ranged from 0.69 for digestive (SD 1.01) to 1.52 for fatigue (SD 0.89) symptom domains. Pearson and Spearman correlations between each domain score and total domain score showed that pain and fatigue symptom scores were highly correlated (*r* > 0.7) with the activity impacts score. At time point 1, a floor effect was observed for neuropathy, digestive, and cognitive symptom domain scores and the social impact domain score. A domain score of 0, the lowest possible score, was detected in ≥ 40% of patients for neuropathy, digestive, and cognitive symptoms and social impact domain scores. No ceiling effect was observed for any of the domain scores as fewer than 5% of patients had a score of > 3 (with 4 being the maximum possible score).

### Confirmatory factor analysis

#### Evaluation of the factor structure

**Fig. 2:**
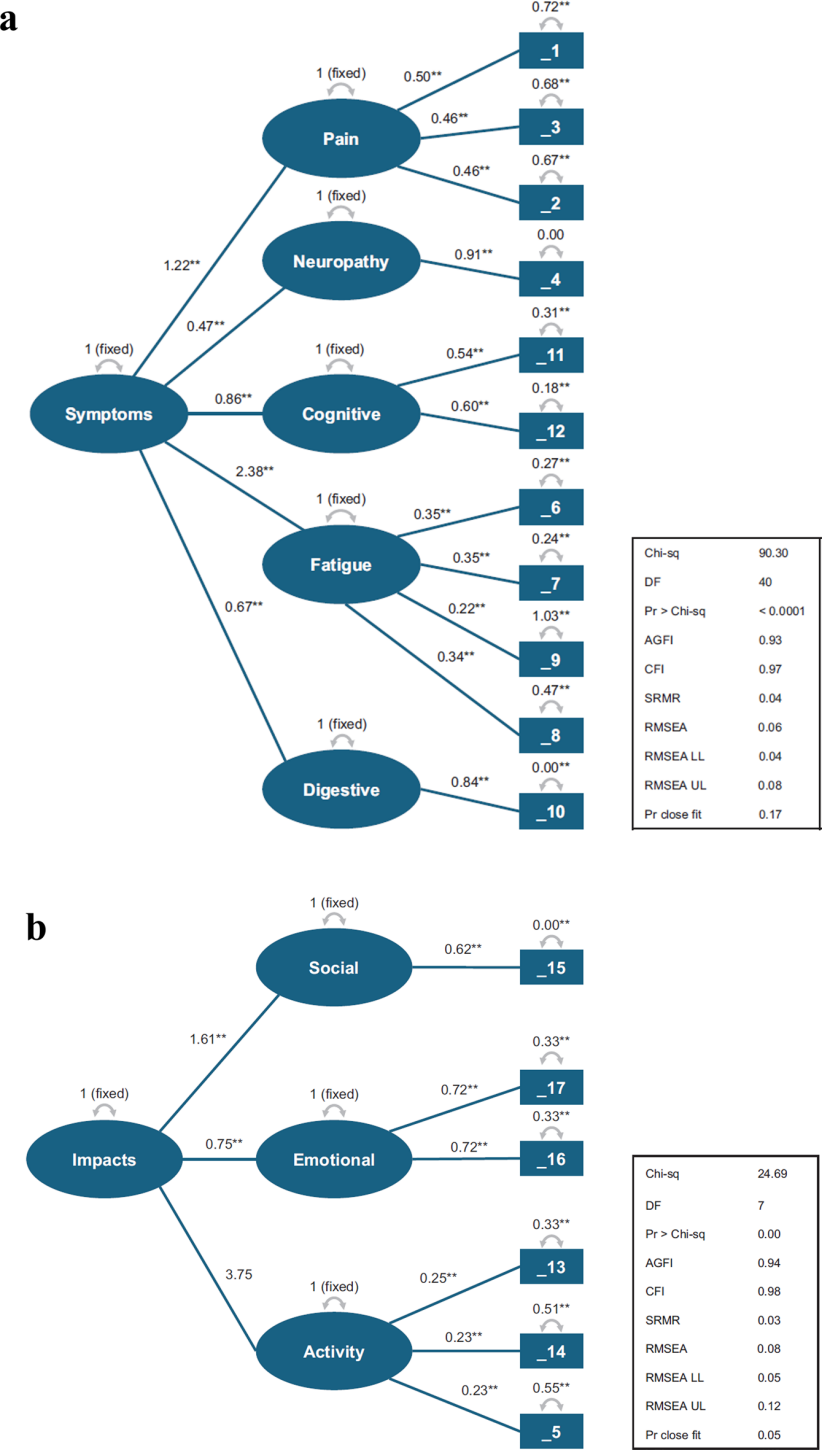
Confirmatory factor analysis final model (Model 2) with second-order free loadings. **a** Model 2 for MySIm-Q symptom-related constructs. **b** Model 2 for MySIm-Q impact-related constructs. 1 indicates worst pain in your back, 2 = worst pain in your legs, 3 = worst pain in areas other than your back or legs, 4 = worst numbness/ tingling in hands/feet, 5 = pain interferes with daily activities, 6 = have low energy, 7 = tire easily, 8 = experience muscle weakness, 9 = trouble with your sleep, 10 = poor appetite, 11 = difficulty with your memory, 12 = difficulty concentrating on things, 13 = limited in doing daily activities, 14 = difficulty walking, 15 = limited in your social life, 16 = felt depressed about multiple myeloma, 17 = worry multiple myeloma could get worse; AGFI = adjusted goodness of fit; CFI = comparative fit index, DF = degrees of freedom, LL = lower limit, MySIm-Q = Multiple Myeloma Multiple Myeloma Symptom and Impact Questionnaire, Pr > Chi-sq = Pearson’s chi-square, RMSEA = root mean square error of approximation, SRMR = standardized root mean square residual, UL = upper limit. **p < 0.01

Confirmatory factor analysis showed that for the total symptom domain score, both second-order models (Model 2 and Model 3) fit the data well, with Model 2 as the best-fitting model (Fig. 2a). Models 2 and 3 had acceptable fit with root mean square error of approximation (RMSEA) < 0.08 and a comparative fit index (CFI) > 0.95. For Model 1, RMSEA and CFI were 0.13 and 0.83 for total symptom score and 0.24 and 0.84 for total impact score, respectively. For Model 2, RMSEA and CFI were 0.06 and 0.97 for total symptom score and 0.08 and 0.98 for total impact score. For Model 3, RMSEA and CFI were 0.07 and 0.96 for total symptom score and 0.16 and 0.93 for total impact score, respectively. For Model 2, the standardized root mean square residual value (0.04 for symptom scores and 0.03 for impact scores, respectively) was also below a recommended threshold for acceptable fit (< 0.08) [21] and suggests the fit of Model 2 was acceptable (Fig. 2a and b).

#### Assessment of the correlation between factor scores and observed scores

Factor model-based symptom and impact scores, based on the parameter estimates, were computed for each patient. Then, correlations between each factor score and its corresponding simple observed-based score were computed. Model 2 fit the data well for both symptom and impact scores, with suggested thresholds for acceptable fit being exceeded for adjusted goodness of fit (≥ 0.90) and CFI (≥ 0.95) [21]. The correlation between observed and factor scores estimated from Model 2 was 0.94 for symptom scores and 0.98 for impact scores. Overall, these findings suggest that a simple average of item scores yields the same rank ordering as measures based on the more complex second-order factor analytic model.

#### Reliability

Test-retest reliability estimates were slightly below the prespecified ICC(2,1) threshold of 0.70, with ICC coefficient values of 0.67 and 0.65, respectively, for the MySIm-Q symptom and impact domain scores. The sensitivity analysis in stable patients (i.e. those who have no change in PGIS score) confirmed results observed in the original test-retest reliability analysis. The internal reliability consistency estimates for all MySIm-Q multi-item, symptom and impact domain scores and the MySIm-Q total symptom and total impact domain scores at time point 1 met the preestablished threshold (each overall McDonald’s ω-coefficient was ≥ 0.70 based on the best fitting confirmatory factor analysis model (i.e., Model 2); ω for total symptom score = 0.87, ω for total impact score = 0.79; Supplementary Table 1).

#### Validity

Known-groups validity assessment evidence suggested a monotonic relationship between MySIm-Q domain scores and known groups of increasing PGIS (none/mild vs. moderate vs. severe/very severe) (Supplementary Table 2).

Evidence of item-level convergent validity was obtained. All correlations between each item and their adjusted hypothesized domain were > 0.40 (Supplementary Table 3). Evidence of item-level discriminant validity was also strong for all but three items: worst pain in your back (item 1; adjusted for overlap with pain symptoms); worst pain in your legs (item 2; adjusted for overlap with pain symptoms); and limited in doing daily activities (item 13; adjusted for overlap with activity impacts). These items correlated more highly with another scale than their own hypothesized scale after adjusting for overlap, suggesting it may be worth exploring if grouping some of these items together into the same domain is more appropriate in future research with varied MM patient populations.

Evidence of domain convergent validity was generally positive; the digestive symptom domain was more specific to appetite loss than to nausea and vomiting when assessed by EORTC QLQ-C30 (*r* = 0.79 and 0.41; Table 1). Therefore, domain convergent validity assessment results were inconclusive for the digestive symptom domain. Similarly, domain discriminant validity was mostly supported. However, similar to results of the convergent validity assessment, domain discriminant validity assessment results were inconclusive for the digestive symptom domain, but they were also inconclusive for the dyspnea/shortness of breath items.

**Table 1:** Pearson correlations between MySIm-Q scales and EORTC QLQ-30 measures at time point 1

EORTC QLQ-30	Pain	Neuropathy	Fatigue	Digestive	Cognitive	Activity	Social	Emotional	Total symptoms	Total impact
Physical functioning	−0.55	−0.22	−0.66	−0.41	−0.44	−0.79	−0.63	−0.39	−0.63	−0.72
Role functioning	−0.51	−0.17	−0.64	−0.30	−0.47	−0.73	−0.64	−0.40	−0.58	−0.71
Emotional functioning	−0.33	−0.15	−0.50	−0.25	−0.44	−0.44	−0.43	−0.68	−0.46	−0.61
Cognitive functioning	−0.35	−0.25	−0.51	−0.26	−0.79	−0.44	−0.43	−0.35	−0.59	−0.49
Social functioning	−0.50	−0.23	−0.58	−0.34	−0.48	−0.67	−0.72	−0.44	−0.59	−0.73
Fatigue	0.54	0.21	0.77	0.47	0.50	0.70	0.59	0.46	0.69	0.69
Nausea/vomiting	0.33	0.23	0.40	0.41	0.35	0.37	0.33	0.25	0.48	0.38
Pain	0.74	0.26	0.61	0.34	0.35	0.80	0.61	0.41	0.64	0.72
Dyspnea	0.28	0.12	0.44	0.28	0.37	0.42	0.32	0.29	0.41	0.41
Appetite loss	0.31	0.16	0.45	0.79	0.29	0.41	0.35	0.24	0.57	0.40

EORTC QLQ-30 indicates European Organisation for Research and treatment of cancer quality of life questionnaire; MySIm-Q = Multiple Myeloma Symptom and Impact Questionnaire

## Discussion

Our findings support key measurement properties of the MySIm-Q using data from patients with lenalidomide-refractory MM who received 1 to 3 prior LOT, including a PI and an IMiD, and treated with both SOC regimens and CAR-T therapy. Importantly, our results provide further support for use of the total symptom score and total impact score as clinical outcome assessments in patients with MM [16]. Internal consistency reliability estimates were acceptable for all MySIm-Q symptom and domain scores and both MySIm-Q total symptom and total impact domain scores. Confirmatory factor analysis yielded posited models that fit well with observed data. For the data matrix (i.e., the item responses), the more complex model(s) provided a superior fit to the simpler unidimensional model, which did not fit as well by comparison. As all models necessarily constitute approximations of reality, the most defensible model (i.e., Model 2, the best fitting model) supported by the available evidence was selected. Notably, more parsimonious observed-based scoring was highly correlated with MySIm-Q model–based factor scores. Known-groups validity of both the total symptom domain score and total impact domain score was supported based on our analyses as reflected by discrimination across disease severity groups based on PGIS. Item-level convergent validity was met for all items and item-level discriminant validity was met for nearly all items. Therefore, grouping these items in a similar domain was appropriate in this study. Domain convergent and discriminant validity properties for the total symptom domain score and total impact domain score were largely met.

The collective evidence gathered here suggests that the MySIm-Q instrument can be used as a fit-for-purpose PRO instrument and supports several validity and reliability arguments, including internal consistency and known-group differences. Similarly, preliminary assessment of measurement properties of the MySIm-Q symptom domain was conducted using data from the multicohort CARTITUDE-2 study. CARTITUDE-2 cohort A comprised patients with lenalidomide-refractory MM after 1 to 3 prior LOT, including a PI and IMiD; cohort B comprised patients with 1 prior LOT, including a PI and an IMiD, who had progressive disease ≤ 12 months after autologous stem cell transplant or frontline antimyeloma therapy for patients without autologous stem cell transplant; cohort C comprised patients previously treated with a PI, IMiD, anti-CD38 antibody, and noncellular B-cell maturation antigen–directed therapy (e.g. antibody-drug conjugate and bispecific T-cell engager). Analysis in this patient population showed the MySIm-Q instrument can detect changes in MM symptoms, further supporting its use as a fit-for-purpose PRO instrument in MM clinical trials [16]. Furthermore, our analysis demonstrates that MySIm-Q captures the relevant concepts of novel treatments on MM-related symptoms and impacts experienced by patients and complements existing legacy PRO instruments such as the EORTC QLQ-C30 and EORTC QLQ-MY20 while taking into account the changing treatment landscape in MM [11].

A strength of our study is that it was a rigorous psychometric validation exercise based on a framework outlined by the US Food and Drug Administration. A potential limitation of this study was that test-retest assessments were between 21 and 28 days apart, which is a longer interval than usual (7 days is typical) and could have contributed to slightly lower than expected intraclass correlation coefficient values. The longer the interval, the more likely intercurrent events have the potential to impact the coefficient value as well as violate the assumption of no change between arms at “test” and “retest”, a key assumption of the coefficient of stability. Although intraclass correlation values did not meet our prespecified threshold of ICC(2,1) below 0.70 for acceptable domain test-retest reliability, this is common for domains with relatively few items and when the range of scores is somewhat limited, as in the MySIm-Q. However, similar analyses from the CARTITUDE-2 study demonstrated adequate test-retest reliability, supporting its reliability and reproducibility as a PRO instrument [16]. This suggests the need to replicate this analysis with additional MM cohorts in future research.

Given the psychometric properties of any measurement tool reflect characteristics of both its items as well as the patients completing the instrument, it is critical to undertake additional research with varied cohorts to assess the generalizability of findings. As such, the MySIm-Q PRO continues to be investigated in cohorts who are being treated with bispecific antibodies, such as teclistamab and talquetamab [22, 23], underscoring the global applicability of the instrument in patients with MM receiving immunotherapies.

## Conclusions

These results demonstrate that MySIm-Q yields reliable and valid scores based on a number of evidentiary sources, supporting its use as a fit-for-purpose PRO instrument in clinical outcome assessments for patients with MM. The MySIm-Q contributes to the database of existing PRO measures while addressing the limitations of legacy PRO instruments, with corroborating reliability and validity evidence. Additional evidence of reliability and validity in additional multiple myeloma samples is ongoing, as well as results to support the ability to detect change and interpretation of scores with meaningful change thresholds.

## Supplementary Information

Below is the link to the electronic supplementary material.

Supplementary Material 1

## Data Availability

The data sharing policy of Janssen Pharmaceutical Companies of Johnson & Johnson is available at https://www.janssen.com/clinical-trials/transparency. As noted on this site, requests for access to the study data can be submitted through Yale Open Data Access (YODA) Project site at http://yoda.yale.edu.
