# Supplementary material for: Validation of the Multiple Myeloma Symptom and Impact Questionnaire (MySIm-Q) in patients with multiple myeloma who were enrolled in the CARTITUDE-4 trial
Source: J Patient Rep Outcomes. 2026 Mar 13;10:64. doi: 10.1186/s41687-026-01034-z (PMC13100226; doi:10.1186/s41687-026-01034-z)
Supplement: Supplementary file 1 — Supplementary Material 1 [file 41687_2026_1034_MOESM1_ESM.docx]

**Validation of the Multiple Myeloma Symptom and Impact Questionnaire (MySIm-Q) in Patients With Multiple Myeloma Who Were Enrolled in the CARTITUDE-4 Trial**

**SUPPLEMENTARY INFORMATION**

**Table of Contents Page**

**Supplementary Methods 3**

*PRO instruments in CARTITUDE-4* 3

*PRO measurement collection* 3

**Supplementary Tables 5**

*Supplementary Table 1: Internal consistency reliability of MySIm-Q scales at
time point 1* 5

*Supplementary Table 2: Known-groups analysis of MySIm-Q scores by
PGIS scores at time point 1* 6

*Supplementary Table 3: Item-level convergent and discriminant validity of
MySIm-Q scales at time point 1* 7

**Supplementary References 9**

**Supplementary Methods**

***PRO instruments in CARTITUDE-4***

In addition to the MySIm-Q, additional PRO instruments used in CARTITUDE-4 included the following:

- EORTC QLQ-C30: a cancer-specific PRO questionnaire widely used in patients with MM.^1-6^ It is composed of a GHS/QoL scale, five functional scales (physical, role, emotional, cognitive, and social), and three symptom scales (fatigue, nausea and vomiting, and pain). Patients rate items based on the extent to which health challenges have impacted them during the past week on a four-point rating scale (not at all [1], a little [2], quite a bit [3], and very much [4]). Scores range from 0 to 100, with high scores representing better functioning or worse symptoms.
- PGIS: a single item used to assess a patient’s perception of the severity of their MM using a five-point rating scale (none [1], mild [2], moderate [3], severe [4], and very severe [5]).

***PRO measurement collection***

During the first approximately 12 weeks of the study, PRO measurement collection varied by study treatment (based on study administration schedule differences):

- In the SOC arm (21-day PVd cycles or 28-day DPd cycles), PROs were collected on day 1 of cycle 1 and every 3 to 4 weeks for weeks 1 to 12.
- In the cilta-cel arm, PROs were collected at three time points prior to infusion (within 72 hours of apheresis, on day 1 of first cycle of bridging therapy, and on day 1 of conditioning) and 28 days after infusion (approximately week 12).

Subsequently, the days of PRO collection were matched across study arms and occurred every 12 weeks until week 40 and then every 24 weeks thereafter.

**Supplementary Table 1** Internal consistency reliability of MySIm-Q scales at time point 1

| **Items** | **λ (Lambda)** | **ψ (Psi)** | **R²** |
| --- | --- | --- | --- |
| **Symptoms – Model 2 unstandardized** |  |  |  |
| Pain symptoms |  |  |  |
| 1. Worst pain in your back | 0.4997 | 0.7183 | 0.4640 |
| 2. Worst pain in your legs | 0.4598 | 0.6737 | 0.4387 |
| 3. Worst pain in areas other than your back or legs | 0.4608 | 0.6794 | 0.4377 |
| Neuropathy |  |  |  |
| 4. Worst numbness/tingling in hands/feet | 0.9099 | 0.0047 | 0.9954 |
| Fatigue symptoms |  |  |  |
| 6. Have low energy | 0.3530 | 0.2722 | 0.7526 |
| 7. Tire easily | 0.3545 | 0.2401 | 0.7767 |
| 8. Experience muscle weakness | 0.3432 | 0.4719 | 0.6239 |
| 9. Trouble with your sleep | 0.2221 | 1.0331 | 0.2408 |
| Digestive |  |  |  |
| 10. Poor appetite | 0.8422 | 0.0000 | 1.0000 |
| Cognitive symptoms |  |  |  |
| 11. Difficulty with your memory | 0.5366 | 0.3056 | 0.6222 |
| 12. Difficulty concentrating on things | 0.6040 | 0.1781 | 0.7817 |
| **Total symptom score** | 5.5859 | 4.5771 | – |
| **Omega** | 0.8721 | | |
| **Impact – Model 2 unstandardized** |  |  |  |
| Activity impacts |  |  |  |
| 5. Pain interferes with daily activities | 0.2280 | 0.5511 | 0.5874 |
| 13. Limited in doing daily activities | 0.2488 | 0.3283 | 0.7400 |
| 14. Difficulty walking | 0.2281 | 0.5058 | 0.6083 |
| Social Functioning |  |  |  |
| 15. Limited in your social life | 0.6244 | 0.0000 | 1.0000 |
| Emotional impacts |  |  |  |
| 16. Felt depressed about multiple myeloma | 0.7160 | 0.3274 | 0.7097 |
| 17. Worry multiple myeloma could get worse | 0.7211 | 0.3301 | 0.7109 |
| **Total impact score** | 2.7663 | 2.0427 | – |
| **Omega** | 0.7893 | | |

MySIm-Q indicates Multiple Myeloma Symptom and Impact Questionnaire.

**Supplementary Table 2** Known-groups analysis of MySIm-Q scores by PGIS scores at time point 1

| **Score** | **PGIS, mean (SD)** | | | ***p-*value** | | |
| --- | --- | --- | --- | --- | --- | --- |
|  | **None/mild**  **n = 87** | **Moderate**  **n = 154** | **Severe/very severe**  **n = 118** | **None/mild vs moderate** | **None/mild vs severe/very severe** | **Moderate vs severe/very severe** |
| Total symptom score | 0.57 (0.47) | 0.97 (0.55) | 1.31 (0.74) | < 0.0001 | < 0.0001 | < 0.0001 |
| Pain symptoms | 0.57 (0.63) | 1.05 (0.75) | 1.51 (1.01) | < 0.0001 | < 0.0001 | < 0.0001 |
| Neuropathy symptoms | 0.55 (0.79) | 0.79 (0.89) | 1.14 (1.19) | 0.0428 | < 0.0001 | 0.0049 |
| Fatigue symptoms | 0.93 (0.68) | 1.55 (0.81) | 1.93 (0.89) | < 0.0001 | < 0.0001 | 0.0003 |
| Digestive symptoms | 0.34 (0.73) | 0.67 (0.95) | 0.97 (1.19) | 0.0063 | < 0.0001 | 0.0228 |
| Cognitive symptoms | 0.87 (0.74) | 1.42 (0.91) | 1.94 (1.00) | < 0.0001 | < 0.0001 | < 0.0001 |
| Total impact score | 0.55 (0.50) | 1.20 (.76) | 1.75 (0.94) | < 0.0001 | < 0.0001 | < 0.0001 |
| Activity impacts | 0.41 (0.51) | 1.08 (0.91) | 1.63 (1.07) | < 0.0001 | < 0.0001 | < 0.0001 |
| Social impacts | 0.36 (0.75) | 1.11 (1.07) | 1.69 (1.27) | < 0.0001 | < 0.0001 | < 0.0001 |
| Emotional impacts | 0.87 (0.74) | 1.42 (0.91) | 1.94 (1.00) | < 0.0001 | < 0.0001 | < 0.0001 |

*p-*values are from a two-sample test.

MySIm-Q indicates Multiple Myeloma Symptom and Impact Questionnaire; PGIS = patient global impression of severity, SD = standard deviation

**Supplementary Table 3** Item-level convergent and discriminant validity of MySIm-Q scales at time point 1

|  | **Pain** | **Neuro-pathy** | **Fatigue** | **Digestive** | **Cognitive** | **Activity** | **Social** | **Emotional** | **Total symptom** | **Total impact** | **Adjusted for overlap** |
| --- | --- | --- | --- | --- | --- | --- | --- | --- | --- | --- | --- |
| Pain symptoms | | | | | | | | | | | |
| 1. Worst pain in your back | 0.81 | 0.20 | 0.49 | 0.27 | 0.28 | 0.63 | 0.46 | 0.35 | – | – | 0.54 |
| 2. Worst pain in your legs | 0.78 | 0.39 | 0.47 | 0.23 | 0.26 | 0.57 | 0.42 | 0.25 | – | – | 0.51 |
| 3. Worst pain in areas other than your back or legs | 0.79 | 0.32 | 0.45 | 0.27 | 0.31 | 0.51 | 0.48 | 0.19 | – | – | 0.53 |
| Neuropathy symptoms | | | | | | | | | | | |
| 4. Worst numbness/ tingling in hands/feet | 0.38 | 1.00 | 0.36 | 0.20 | 0.27 | 0.32 | 0.26 | 0.19 | – | – |  |
| Fatigue symptoms | | | | | | | | | | | |
| 6. Have low energy | 0.49 | 0.29 | 0.87 | 0.47 | 0.47 | 0.68 | 0.57 | 0.46 | – | – | 0.76 |
| 7. Tire easily | 0.50 | 0.31 | 0.88 | 0.43 | 0.46 | 0.66 | 0.59 | 0.47 | – | – | 0.77 |
| 8. Experience muscle weakness | 0.55 | 0.30 | 0.83 | 0.44 | 0.48 | 0.68 | 0.53 | 0.47 | – | – | 0.68 |
| 9. Trouble with your sleep | 0.39 | 0.25 | 0.69 | 0.30 | 0.34 | 0.44 | 0.42 | 0.31 | – | – | 0.44 |
| Digestive symptoms | | | | | | | | | | | |
| 10. Poor appetite | 0.32 | 0.20 | 0.50 | 1.00 | 0.35 | 0.47 | 0.39 | 0.35 | – | – |  |
| Cognitive symptoms | | | | | | | | | | | |
| 11. Difficulty with your memory | 0.28 | 0.24 | 0.47 | 0.32 | 0.92 | 0.38 | 0.42 | 0.32 | – | – | 0.70 |
| 12. Difficulty concentrating on things | 0.37 | 0.26 | 0.52 | 0.32 | 0.92 | 0.49 | 0.51 | 0.40 | – | – | 0.70 |
| Activity impacts | | | | | | | | | | | |
| 5. Pain interferes with daily activities | 0.74 | 0.30 | 0.65 | 0.34 | 0.31 | 0.87 | 0.61 | 0.45 | – | – | 0.71 |
| 13. Limited in doing daily activities | 0.57 | 0.27 | 0.70 | 0.45 | 0.54 | 0.87 | 0.73 | 0.46 | – | – | 0.71 |
| 14. Difficulty walking | 0.57 | 0.26 | 0.62 | 0.44 | 0.41 | 0.88 | 0.63 | 0.36 | – | – | 0.73 |
| Social impacts | | | | | | | | | | | |
| 15. Limited in your social life | 0.57 | 0.26 | 0.64 | 0.39 | 0.51 | 0.75 | 1.00 | 0.46 | – | – |  |
| Emotional impacts | | | | | | | | | | | |
| 16. Felt depressed about multiple myeloma | 0.33 | 0.18 | 0.47 | 0.31 | 0.35 | 0.44 | 0.44 | 0.92 | – | – | 0.71 |
| 17. Worry multiple myeloma could get worse | 0.29 | 0.18 | 0.50 | 0.33 | 0.37 | 0.45 | 0.42 | 0.93 | – | – | 0.71 |
| Total symptom score | | | | | | | | | | | |
| Pain symptoms | – | – | – | – | – | – | – | – | 0.73 | 0.64 | 0.57 |
| Neuropathy symptoms | – | – | – | – | – | – | – | – | 0.64 | 0.30 | 0.39 |
| Fatigue symptoms | – | – | – | – | – | – | – | – | 0.83 | 0.76 | 0.71 |
| Digestive symptoms | – | – | – | – | – | – | – | – | 0.68 | 0.47 | 0.45 |
| Cognitive symptoms | – | – | – | – | – | – | – | – | 0.68 | 0.55 | 0.51 |
| Total impact score | | | | | | | | | | | |
| Activity impacts | – | – | – | – | – | – | – | – | 0.76 | 0.88 | 0.73 |
| Social impacts | – | – | – | – | – | – | – | – | 0.65 | 0.89 | 0.70 |
| Emotional impacts | – | – | – | – | – | – | – | – | 0.50 | 0.75 | 0.51 |

Correlations were adjusted for overlap of each item/scale based on a derivation of the particular score where that item was excluded.

MySIm-Q indicates Multiple Myeloma Symptom and Impact Questionnaire.

**Supplementary References**

1. Ojo AS, Araoye MO, Ali A, Sarma R. (2024). The impact of current therapeutic options on the health-related quality of life of patients with relapse/refractory multiple myeloma: a systematic review of clinical studies. *J Cancer Surviv,* *18*(3), 673-697.
2. Wang T, Lu Q, Lang L. (2023). Assessment tools for patient-reported outcomes in multiple myeloma. *Support Care Cancer,* *31*(7), 431.
3. Aaronson NK, Ahmedzai S, Bergman B, et al. (1993). The European Organization for Research and Treatment of Cancer QLQ-C30: a quality-of-life instrument for use in international clinical trials in oncology. *J Natl Cancer Inst,* *85*(5), 365-376.
4. Iravani K, Jafari P, Akhlaghi A, Khademi B. (2018). Assessing whether EORTC QLQ-30 and FACT-G measure the same constructs of quality of life in patients with total laryngectomy. *Health Qual Life Outcomes, 16*(1), 183.
5. Wisløff F, Eika S, Hippe E, et al. (1996). Measurement of health-related quality of life in multiple myeloma. Nordic Myeloma Study Group. *Br J Haematol, 92*(3), 604-613.
6. Wisløff F, Hjorth M. Health-related quality of life assessed before and during chemotherapy predicts for survival in multiple myeloma. Nordic Myeloma Study Group. (1997). *Br J Haematol, 97*(1), 29-37.
